# Fracture of Ceramic Liner and Head in a Total Hip Arthroplasty with a Sandwich Type Cup

**DOI:** 10.1155/2013/291691

**Published:** 2013-04-18

**Authors:** Diego Reátegui, Sebastián García, Guillem Bori, Xavier Gallart

**Affiliations:** Orthopaedic Department, Hospital Clínic de Barcelona, Stair 12, 4th floor, Villarroel 170, 08036 Barcelona, Spain

## Abstract

Due to its advantages, ceramic-on-ceramic bearings have been widely used in young patients for almost 30 years. Long-term survivorship, low wear, and low biological reactivity to particles are some of its characteristics. Even though this material has had a lot of improvements, the risk of fracture is one of the concerns. There have been reports of fracture of ceramic in the acetabular liner and head but no fractures of both in the same patient. We report a case of a fracture in a sandwich type acetabular liner and the ceramic head in a patient involving ankylosing spondylitis. It occurred three years after the operation and with no history of direct trauma. We decided to change the bearing surfaces to metal polyethylene without removing the metal back. The patient is satisfied by the clinical results after a 5-year followup.

## 1. Introduction

Ceramic-on-ceramic bearing (CoC) in total hip arthroplasty (THA) was developed in the early 1970s [1] and has been used for almost 40 years with excellent results [2, 3]. It was introduced to reduce wear and to increase long-term survivorship [4]. Some of its advantages are scratch resistance, low wear, wettable surface, and low biological reactivity to wear particles [5]. Nevertheless it also has disadvantages; one of the major concerns of the CoC in THA is the risk of fracture due to its brittleness. Some authors have reported fractures of the ceramic head [6–11] others reported fracture of the ceramic acetabular insert [12–14], but there is no report of fracture of both.

We report a case of a fracture of the ceramic acetabular liner and the ceramic head. We discuss its causes and possible treatments.

## 2. Case Report

In October 2004, a 35-year-old male with a history of ankylosing spondylitis, right THA in 1999, and a body mass index (BMI) of 25,5 kg/m^2^ underwent a left THA because of osteoarthritis. A direct lateral approach was used. An Alloclansic SL Stem (Zimmer, Warsaw, IN, USA), a 28 mm, alumina ceramic head, a 52 mm, Allofit-S. Cup (Zimmer, Warsaw, Indiana USA) with a sandwich ceramic liner were implanted. The metal shell was fixed with one screw to ensure fixation. During intraoperative tests, no impingement was seen. The inclination of the cup measured in postoperative radiographs was 44°. The postoperative course was uneventful, and the patient was discharged 6 days later. 

**Figure 1:**
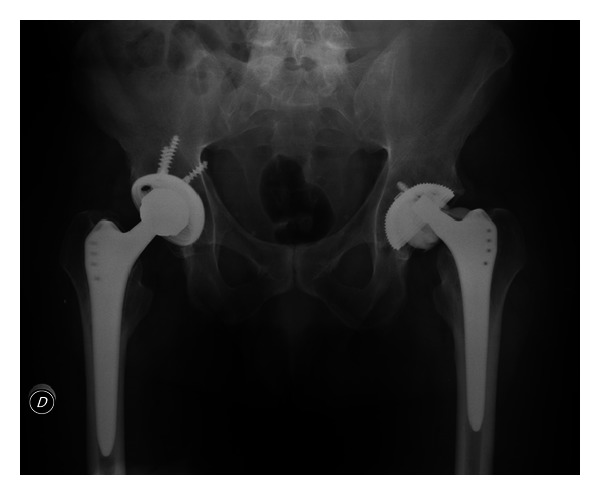
Radiograph showing fracture of the ceramic acetabular insert and the ceramic head.

**Figure 2:**
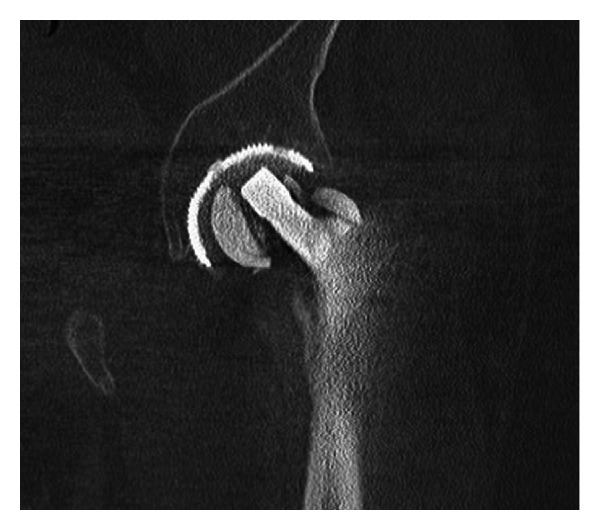
Tomography scan confirmed damage of the ceramic head component.

The patient was asymptomatic for three years after surgery until he returned in May 2007 through the emergency room with a history of a crunch and sudden pain in the left hip after standing from the sitting position; he denied history of direct trauma to the hip. The radiograph showed fracture of the ceramic acetabular insert and the ceramic head (Figure 1). CT scan confirmed damage of the head (Figure 2). No signs of loosening of the stem were shown in the radiograph or CT scan. Cross-sectional images through femur showed integration of the stem on both anterior and posterior wall. 

**Figure 3:**
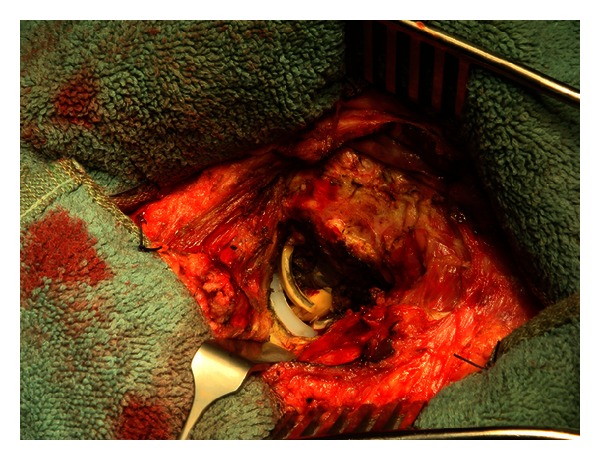
Signs of metallosis, comminuted fracture of the ceramic head, loosening, and fracture of the sandwich ceramic liner.

A revision surgery was performed two days after hospital admission. Findings were signs of metallosis, comminuted fracture of the ceramic head, loosening, and fracture of the sandwich ceramic liner (Figure 3). All fragments were removed performing an extended capsulectomy in order to clean the joint space. The metal back had 5° of anteversion. We decided not to remove the cup and changed the head to a cobalt-chrome alloy head and inserted a cross-linked polyethylene liner. The femoral stem was left in its place. All intraoperative cultures were negative. There were no postoperatory complications. Patient went home one week after surgery. We are satisfied by the clinical and radiological results after a 5-year followup.

## 3. Discussion

CoC was introduced in total hip arthroplasty to reduce wear and to increase long-term survival. D'Antonio et al. [4] found in a prospective randomized study, with a minimum of 10-year followup, that there was no difference between the control group (metal-on-polyethylene) and the alumina bearing couple cohorts with regard to bearing related failures. But a higher rate revision surgery occurred in the control group, 10, 5% versus 3.1%, for other reasons such as osteolysis, loosening of component, dislocation, and infection.

There have been improvements in the mechanical properties of ceramic materials such as hot isostatic pressing, laser marking, and nondestructive proof testing [1]. Nevertheless one of the problems in ceramic bearing is the risk of fracture. Most of these fractures associated with contemporary third generation ceramic material occurred in the ceramic liner, with or without use of sandwich type acetabular components such as the one in our case [15, 16]. These types of ceramic cup with a polyethylene backing (sandwich cups) were developed to act as a shock absorber, although some authors suggest that thickness of the ceramic insert (only 4 mm) would be a negative factor that contributes to fractures [14]. Other authors suggest that the mismatch properties of these compounds (polyethylene: hydrophobic, ceramic: hydrophilic) could create an aqueous environment and micromovements between cup components [13]. 

Patient risk factors contributing to ceramic component fracture include age, activities and a history of trauma [12, 14], there are no cases describing fractures of components in patients with CoC and ankylosing spondylitis, although there is much literature that describes worse outcomes and functional prognosis in THA in these patients [17]. Some reports also attribute ceramic liner fracture in Asian population due to activities such as squatting, kneeling, and sitting crossed legged which can cause impingement and liner fracture [13]. Our patient was slightly overweighted and reported no history of trauma but had an important comorbidity.

Most important risks to ceramic bearing fracture are, according to Barrack et al. [7], intraoperative factors such an optimal component positioning. They describe that vertical cup positioning placed at 60° had higher wear rates than the ones placed at the optimal 45°. They concluded that acetabular cup angles exceeding 55° showed more wear than those placed in optimal positioning [7]. Acetabular components should be placed at less than 45° abduction and 10°–15° anteversion in order to reduce wear by distributing forces over the greatest amount of surface between the head and cup [5, 7, 12–14]. In our case the only variable that did not match these parameters was the cup anteversion, only of 5°; could be an important but not definitive contributing factor that might have caused a stress concentration at the head and acetabular rim. The real cause of fracture in our case is unknown. We think that probably there was a fracture of one component that was not noticed by the patient who continued bearing weight until finally the other component failed.

There has been a lot of discussions and controversies regarding the type of bearing that should be used after a fracture in CoC, but there are no prospective studies due to the few patients having this complication. Some authors highly recommend hard bearings such as ceramic on ceramic or metal on metal. They suggest that soft bearings such as polyethylene or cross-linked polyethylene may be vulnerable to wear by retained ceramic particles. Some authors think that another ceramic liner would be more resistant to wear of the retained particles but that an unrecognized defect on the acetabular shell may lead to a new fracture [12]. Removal of all the components and use of an alumina-on-alumina or ceramic-on-polyethylene bearing has been described too [18]. Although this last option carries out danger of damage to bone stock and blood loss. In our case we decided to change to metal-polyethylene bearing after meticulous debridement, with good results.

We conclude that CoC should be used in THA especially in young patients due to its long-term survival rates and low rates of revision surgery. But a correct positioning of ceramic compound (abduction, anteversion, and femoral stem angle) should be obtained in order to avoid a serious complication such as fracture of the material. Ceramic liner thickness must be chosen correctly depending on type of cup. Patient comorbidities should also be taken into consideration when choosing CoC in THA. Finally, the best option bearing surface to change after a fracture in CoC is not defined yet, and we believe that metal-polyethylene bearing with an additional extended capsulectomy could be a good choice.
